# Mechanisms of disease: inflammasome activation and the development of type 2 diabetes

**DOI:** 10.3389/fimmu.2013.00050

**Published:** 2013-03-08

**Authors:** Ryan W. Grant, Vishwa D. Dixit

**Affiliations:** Immunobiology Laboratory, Pennington Biomedical Research Center, Louisiana State University SystemBaton Rouge, LA, USA

**Keywords:** inflammation, T cells, adipocytes, glyburide, macrophages, pycard, IL-1β, caspase-1 apoptosis

## Abstract

Over the recent past, the importance of aberrant immune cell activation as one of the contributing mechanisms to the development of insulin-resistance and type 2 diabetes (T2D) has been recognized. Among the panoply of pro-inflammatory cytokines that are linked to chronic metabolic diseases, new data suggests that interleukin-1β (IL-1β) may play an important role in initiating and sustaining inflammation-induced organ dysfunction in T2D. Therefore, factors that control secretion of bioactive IL-1β have therapeutic implications. In this regard, the identification of multiprotein scaffolding complexes, “inflammasomes,” has been a great advance in our understanding of this process. The secretion of bioactive IL-1β is predominantly controlled by activation of caspase-1 through assembly of a multiprotein scaffold, “inflammasome” that is composed of NLRP3 (nucleotide-binding domain, leucine-rich-containing family, pyrin domain-containing-3) ASC (apoptosis associated speck-like protein containing a CARD) and procaspase-1. The NLRP3 inflammasome appears to be an important sensor of metabolic dysregulation and controls obesity-associated insulin resistance and pancreatic beta cell dysfunction. Initial clinical “proof of concept” studies suggest that blocking IL-1β may favorably modulate factors related to development and treatment of T2D. However, this potential therapeutic approach remains to be fully substantiated through phase-II clinical studies. Here, we outline the new immunological mechanisms that link metabolic dysfunction to the emergence of chronic inflammation and discuss the opportunities and challenges of future therapeutic approaches to dampen NLRP3 inflammasome activation or IL-1β signaling for controlling type 2 diabetes.

## Introduction

With a disease rate of 8.3% and cost of $174 billion, there is no debate that diabetes is a highly prevalent and costly life-long disease (Dall et al., 2010; Centers for Disease Control and Prevention, 2011). Whereas type 1 diabetes is characterized by autoimmune destruction of pancreatic islets, Type 2 diabetes (T2D) has been described as an autoinflammatory disorder, characterized first by insulin resistance in peripheral tissues followed by beta cell failure, including decreased islet size and insulin production (McGonagle and McDermott, 2006).

T2D is clearly associated with obesity, and clinical progression of this disease has been linked to chronic low-grade inflammation due to activation of immune cells. However, up until recently, the identity of specific immunological sensors that are triggered in response to metabolic dysfunction to produce a state of inflammation was not fully understood. The underlying clinical rationale to identify the immunological triggers of metabolically driven inflammation has been to develop approaches to therapeutically target the immune sensors and break the feed-forward cycle of organ dysfunction and development of diabetes. Among several sites of inflammation in metabolic diseases, adipose tissue is a large contributor to circulating proinflammatory cytokines during obesity. Initially, tumor necrosis factor-α (TNFα) was considered a possible therapeutic target because its adipose tissue expression was increased in multiple rodent-obesity models and TNFα decreased insulin signaling in insulin sensitive tissues (Hotamisligil et al., 1993, 1994a,b; Hotamisligil and Spiegelman, 1994). Accordingly, *Tnf* mRNA expression was shown to be increased in adipose tissue of obese hyperinsulinemic human subjects (Hotamisligil et al., 1995). Furthermore, weight loss-induced improvement in insulin-sensitivity was associated with reduction in TNF suggesting that this pro-inflammatory cytokine impairs insulin-action. (Hotamisligil et al., 1995). Consistent with these clinical findings, mechanistic studies using *Tnf*α gene knockout mice or neutralization of TNF with antibodies improved glycemia in obese mice and rats, respectively, making it a potential therapeutic target for T2D (Hotamisligil et al., 1994a; Uysal et al., 1997). Despite overwhelming evidence in favor of TNF having a critical role in regulating inflammation and insulin-action (Hotamisligil et al., 1993, 1994a,b, 1995; Hotamisligil and Spiegelman, 1994; Peraldi et al., 1996; Uysal et al., 1997; Liu et al., 1998), the translation of basic research findings with TNF targeted neutralization approaches to diabetes care in humans has had disappointing results with both acute and chronic treatment (Ofei et al., 1996; Paquot et al., 2000; Di Rocco et al., 2004; Wascher et al., 2011). Further studies to enhance the delivery and tissue availability of TNF targeted treatments are being pursued to improve treatment outcomes. Here, we discuss clinically relevant, novel “inflammasome” mechanisms that regulate interleukin-1β (IL-1β) and IL-18 driven pro-inflammatory cascades. We discuss the current experimental evidence and potential future therapeutic strategies to target the “inflammasome” pathway in prevention and treatment of diabetes.

## The NLRP3 inflammasome regulates IL-1β secretion during metabolic stress

**Figure 1:**
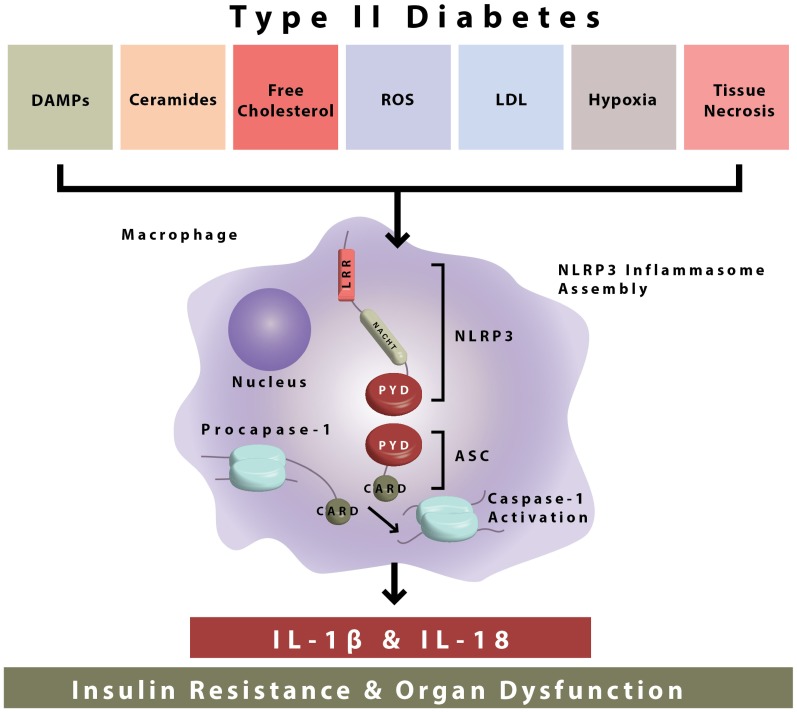
**NLRP3 inflammasome activation during type II diabetes.** The NLRP3 inflammasome is composed of NLRP3, ASC, and procaspase-1. During type II diabetes there is increased accumulation of endogenous danger-associated molecular patterns (ceramides, free cholesterol, etc.) which are sensed by the NLRP3 inflammasome. After sensing of DAMPs, inflammasome assembly occurs by interactions in the protein domains of NLRP3 ASC and procaspase-1. This leads to caspase-1 activation and the secretion of bioactive IL-1β and IL-18, which go on to cause insulin resistance and organ dysfunction.

Given the recent developments in understanding inflammation as a mediator of disease progression, an understanding of factors related to IL-1β regulation is in order. IL-1β is a proinflammatory cytokine that is implicated in the pathogenesis of many inflammatory diseases including diabetes, rheumatoid arthritis and genetic auto-inflammatory disorders (Dinarello, 2011). Although IL-1β is produced by many cell types, it is predominately produced by monocytes, macrophages, and neutrophils (Dinarello, 2011). While most cytokines are regulated at the transcriptional (gene regulation) level, IL-1β is further regulated at the protein level, being stored as an inactive pro-form, which must be cleaved by the IL-1β processing cysteine protease, caspase-1 (Figure 1) for its secretion and activation (Dinarello, 2011). As an additional level of control, caspase-1 is also stored in an inactive state which in turn is activated within large cytosolic multiprotein complexes termed “inflammasomes” upon receiving specific signals (Schroder and Tschopp, 2010). Classically, the inflammasome driven caspase-1 activation and IL-1β secretion occurs as innate immune cells like macrophages engulf bacterial, fungal, and viral proteins. The inflammasome activation is therefore a vital immune response to protect the host against numerous pathogens (Schroder and Tschopp, 2010). Interestingly, new evidence suggest that inflammasome activation may be important in chronic diseases such as obesity and diabetes where low grade inflammation occurs without overt infection (Schroder and Tschopp, 2010).

The NLRP3 inflammasome is formed through the interaction of several cellular proteins. As demonstrated, these key proteins are identified as NLRP3 (for nucleotide-binding domain, leucine-rich-containing family, pyrin domain-containing-3) ASC (apoptosis associated speck-like protein containing a CARD), and procaspase-1 (Figure 1). NLRP3 is expressed predominantly in circulating monocytes and tissue macrophages (Guarda et al., 2011). The NLRP3 inflammasome components contain conserved protein domains and interaction of these domains on inflammasome proteins (i.e., homotypic protein–protein interactions) leads to inflammasome assembly. Thus, the pyrin domain of NLRP3 interacts with the pyrin domain of ASC, and the CARD (caspase activation recruitment domain) of ASC interacts with the CARD domain of procaspase-1 (Figure 1). In this way, the NLRP3 inflammasome is formed leading to the cleavage of procaspase-1 to its enzymatically activated form (Figure 1).

Activation of the NLRP3 inflammasome by bacteria that produce pore forming toxins (Mariathasan et al., 2006), viruses (Muruve et al., 2008; Allen et al., 2009), and fungi (Gross et al., 2009) and the resultant release of IL-1β and IL-18 plays a critical role in host defense. Interestingly, the NLRP3 inflammasome can also be activated in response to accumulation of endogenous damage associated molecular patterns (DAMPs) that are of non-microbial origin and cause “sterile inflammation” (Figure 1). NLRP3 inflammasome activating metabolic “danger signals” include, urate, cholesterol crystals, extracellular ATP, certain fatty acids and islet amyloid peptides (Figure 1). Growing recognition of the NLRP3 inflammasome pathway in triggering sterile inflammation, i.e., inflammation of non-infectious origin has put this innate immune sensor at the crossroads of metabolic disease and inflammation (Wen et al., 2012).

The accumulation of DAMPs during chronic inflammatory diseases is hypothesized to contribute to systemic inflammation and disease pathogenesis. During the pathogenesis of T2D, the NLRP3 inflammasome has been proposed to sense and mediate downstream inflammatory events of “glucotoxicity” (Zhou et al., 2010), islet amyloid polypeptide (Masters et al., 2010), lipid intermediates (i.e., ceramides) (Vandanmagsar et al., 2011), and fatty acids (Wen et al., 2011). The mechanism of inflammasome activation that links these events remains ambiguous, but it may be that these danger signals converge on similar signaling pathways resulting in inflammasome activation.

Mitochondrial damage may be the common pathway between these stimuli because reactive oxygen species appear to be necessary for NLRP3 inflammasome activation, and changes in the redox state of the cell may be a common mediator between danger signals and inflammasome activation (Jin and Flavell, 2010). Treatment of macrophages with LPS and ATP leads to increased reactive oxygen species, mitochondrial damage, and release of mtDNA, a DAMP, into the cytosol (Nakahira et al., 2011). Although mitochondrial DNA is sensed by the AIM2 inflammasome, it also serves as a co-activator of caspase-1 in conjunction with NLRP3 activation by LPS and ATP (Nakahira et al., 2011). Autophagy, a process by which cells remove damaged organelles, buffers inflammasome activation by removing damaged mitochondria, and limiting ROS production and mtDNA escape into the cytosol, and is activated by NLRP3 inflammasome activators (Shi et al., 2012). Moreover, the inflammasome components NLRP3 and ASC are ubiquitinated and subsequently degraded by autophagy, and pro-IL-1β is degraded by autophagy as well (Harris et al., 2011; Shi et al., 2012). Thus, there is a complex interplay between autophagic maintenance of mitochondria and inflammasome proteins that controls inflammasome activation.

Activated caspase-1 proceeds to cleave pro-IL-1β, pro-IL-18, and other undefined substrates. IL-1β signaling then occurs through the IL-1 receptor I (IL1R1), and leads to the activation of the transcription factor nuclear factor-Kappa-Beta (NFkB) and the expression of inflammatory genes (Figure 1) Though lymphocytes are considered a primary target of IL-1β and IL-18, the receptors for both these inflammasome-dependent cytokines are ubiquitously expressed in many different types of cells and tissues, including high expression in pancreatic islets (Boni-Schnetzler et al., 2009). IL-1β signaling is inhibited by IL-1 receptor antagonist (IL-1RA) and decoy receptors, i.e., IL-1R2. Decoy receptors appear to bind IL-1 but do not cause IL-1 signaling. IL-1 signaling is necessary for the inflammatory response, but is highly regulated due to the negative effects of chronic inflammation to body tissues and organs. Regulation of IL-1 signaling is maintained in healthy individuals, but appears to be elevated during chronic proinflammatory disease states, which makes this pathway a valuable therapeutic target in T2D.

## Reduced NLRP3 inflammasome activation increases insulin sensitivity and improves glucose homeostasis

**Figure 2:**
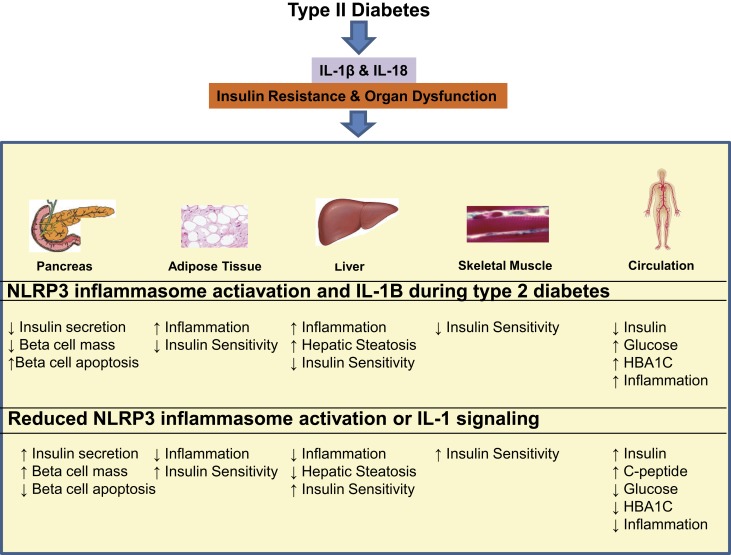
**The consequences of NLRP3 inflammasome activation during type II diabetes and possible benefits of NLRP3 and IL-1 targeted therapies.** NLRP3 inflammasome activation during type II diabetes results in the production of IL-1β and IL-18, which go on to cause insulin resistance and organ dysfunction in the pancreas, adipose tissue, liver, skeletal muscle, and circulation. NLRP3 and IL-1β targeted therapy may have potential to reduce local tissue inflammation and systemic inflammation resulting in systemic improvement in insulin secretion, insulin sensitivity as well as organ function.

The NLRP3 inflammasome, as described in Figure 1, has emerged as a key regulator of glucose and insulin homeostasis. Specifically, pre-clinical studies have shown that the genetic deletion of Nlrp3 and Asc in high-fat diet fed mice results in improved glucose tolerance and enhanced insulin sensitivity (Stienstra et al., 2011; Vandanmagsar et al., 2011; Wen et al., 2011). Along with systemic improvements in glucose and insulin homeostasis, the gene knockout animals, i.e., *Nlrp3*^−/−^ and *Asc*^−/−^ have decreased circulating IL-18, and reduced adipose tissue IL-1β, markers of caspase-1 activation (Vandanmagsar et al., 2011). The pleiotropic effects of inhibition of NLRP3 inflammasome in obesity are evident by improved insulin signaling in adipose tissue, liver, and skeletal muscle and increased insulin secretion in the pancreas [Figure 2; (Stienstra et al., 2011; Vandanmagsar et al., 2011; Wen et al., 2011)].

### Adipose tissue

Adipose is a complex tissue consisting of adipocytes, immune cells, vasculature, and stromal cells. Macrophages are recruited to adipose tissue during obesity and represent the largest population of NLRP3 expressing cells in fat (Weisberg et al., 2003). Recent studies in animal models demonstrate that obesity is associated with progressive caspase-1 activation in adipose tissue (Vandanmagsar et al., 2011). Consistent with the causal role of NLRP3 inflammasome activation in the development of inflammation, deletion of Nlrp3 in mice, prevents obesity-induced caspase-1 activation.

The NLRP3 inflammasome instigates inflammation and causes leukocytosis (i.e., increased immune cell infiltration) in visceral adipose tissue during obesity. Consistent with this, the CD4^+^ and CD8^+^ T cell leukocyte sub populations are specifically reduced in visceral adipose tissue of obese *Nlrp3*^−/−^ mice (Vandanmagsar et al., 2011). This reduction in T cells in response to reduced NLRP3 inflammasome activation is attributable to a decrease in effector-memory T cell subtype presence in the adipose tissue (Vandanmagsar et al., 2011). Together with prior studies, these data suggests that approaches that reduce the number of activated T cell populations in adipose tissue lowers inflammation and improves insulin-action (Feuerer et al., 2009; Nishimura et al., 2009; Winer et al., 2009; Yang et al., 2010).

Visceral adipose tissue macrophages isolated from obese *Nlrp3*^−/−^ mice have reduced expression of the proinflammatory cytokine *Tnf*-α and chemokines (*Ccl20* and *Cxcl1*) involved in lymphocyte recruitment. These results demonstrate the pleiotropic effects of the NLRP3 inflammasome on activation and recruitment of adipose tissue leukocytes. The functional consequences of reduced inflammation in *Nlrp3* and *Asc* deficient mice are improved insulin signaling both in fat and other insulin sensitive tissues (Vandanmagsar et al., 2011; Wen et al., 2011).

Inflammation plays a causal role in insulin resistance, and in rodent models targeting inflammatory cytokine production through genetic and pharmacological approaches results in improvements in insulin signaling (Olefsky and Glass, 2010; Kanneganti and Dixit, 2012). After insulin binds to the insulin receptor, insulin initiates signaling cascades that activate downstream pathways, notably PI3K-AKT and the mitogenic MAP kinase-ERK pathways (Biddinger and Kahn, 2006). In adipose tissue of obese *Nlrp3*^−/−^ mice, phosphorylation of AKT is enhanced indicating greater insulin signaling (Vandanmagsar et al., 2011). The reduction of IL-1 signaling also improves adipose tissue insulin sensitivity in a similar way. Adipose tissue explants from high-fat-fed IL-1 receptor null mice exhibit improved insulin signaling compared to wild type animals, including increased glucose transport, AKT phosphorylation, and increased gene expression of proteins involved with insulin signaling and glucose uptake (*Irs-1* and *Glut4*) (McGillicuddy et al., 2011). These results demonstrate the strong immune and metabolic consequences of NLRP3 inflammasome activation and IL-1 signaling during obesity.

Not only does caspase-1 activation influence whole adipose tissue insulin sensitivity, but it may have direct effects on adipocyte growth, differentiation and metabolism (Stienstra et al., 2010). Interestingly, human and mouse adipocyte cell lines express the caspase-1 protein, and its expression is increased over the course of adipocyte differentiation (Stienstra et al., 2010). Caspase-1 inhibition results in increased expression of favorable adipogenic (*Ppar*γ) and metabolic markers (*Glut4* and *adiponectin*) in 3T3-L1 adipocytes (Stienstra et al., 2010). Given low expression of *Nlrp3* and *Asc* in adipocytes (Vandanmagsar et al., 2011), the significance of adipocyte-derived IL-1β remains ambiguous because macrophages are the predominant cellular sources of IL-1β.

Caspase-1 activates multiple protein substrates other than IL-1β and IL-18, so the exact contribution of downstream mediators of NLRP3 inflammasome activation remains unclear. IL-1β treated 3T3-L1 adipocytes have reduced capacity to differentiate into mature adipocytes, and exhibit insulin resistance and reduce glucose uptake (Lagathu et al., 2006; Jager et al., 2007; Stienstra et al., 2010). Surprisingly, IL-18 does not appear to have an effect on 3T3-L1 adipocyte differentiation or the expression of adipogenic genes in spite of its known pro-inflammatory properties (Stienstra et al., 2010). Given that IL-18 promotes differentiation of T cells into activated pro-inflammatory T-helper1 (T_H_1) IFNγ producing cells (Okamura et al., 1995), it is likely that NLRP3 inflammasome mediated IL-18 secretion induces adipose tissue inflammation via T cell activation (Vandanmagsar et al., 2011; Wen et al., 2011).

### Skeletal muscle and liver

Skeletal muscle is a large metabolically active tissue and accounts for the majority of insulin stimulated glucose disposal. As indicated by improved performance on glucose and insulin tolerance tests, obese *Nlrp3*^−/−^ mice exhibit increased skeletal muscle insulin signaling (Vandanmagsar et al., 2011; Wen et al., 2011). The effects on skeletal muscle are most likely driven through decreased adipose tissue and systemic inflammation because there are not high concentrations of Nlrp3 expressing cells within skeletal muscle, however, local macrophages could be influencing this process.

The liver is also a major contributor to glucose homeostasis by generating glucose through gluconeogenesis. The liver also becomes insulin resistant during the development of T2D, and this is associated with increases in the levels of hepatic steatosis. IL-1β may contribute to this process, given that cultured liver cells treated with IL-1β exhibit decreased insulin response (Nov et al., 2010). *Nlrp3*^−/−^ and *Asc*^−/−^ knockout mice exhibit enhanced liver AKT activation in response to insulin challenge compared to high-fat fed WT mice (Vandanmagsar et al., 2011; Wen et al., 2011). Along with improvements of liver insulin sensitivity, obese *Nlrp3*^−/−^ and *Asc*^−/−^ mice exhibit reduced hepatic steatosis compared to wild type controls (Stienstra et al., 2011; Vandanmagsar et al., 2011). Notably, recent studies also show that NLRP3 inflammasome is required for the maintenance of gut epithelial integrity. In response to methionine-choline deficiency (a model of NASH in mice), NLRP3 inflammasome deficient animals develop exaggerated fatty liver disease due to microbial pathogen-associated molecular patterns leakage into the liver via the portal circulation and activation of the pro-inflammatory response via the Toll-like receptors 4 and 9 (Henao-Mejia et al., 2012). In the same study, *Asc*^−/−^ mice were also noted to have increased weight gain and glucose intolerance during high fat feeding, which were reversible upon administration of antibiotics. This is in contrast with other findings that indicate *Asc*^−/−^ mice have increase insulin sensitivity and glucose tolerance (Youm et al., 2011). T2D has been associated with increased circulating endotoxin concentration, but it is unclear whether this is cause or consequence in disease pathogenesis (Pussinen et al., 2011).

### NLRP3 inflammasome and insulin secretion

Pancreatic islets, macrophages and dendritic cells may all be sources of IL-1β in the pancreas. Cultured pancreatic islets are thought to produce low levels of IL-1β (Arnush et al., 1998). Macrophages and dendritic cells also reside in the pancreas, and macrophages are increased in rodent models of T2D and in patients with T2D (Ehses et al., 2007). It has been shown that IL-1β treatment alone or in combination with IFN-γ induces beta cell death in cell culture, although the exact mechanism by which this occurs is debated (Mandrup-Poulsen et al., 1986; Collier et al., 2011). Consistent with those results, blocking IL-1β action on isolated beta cells using IL-1RA (IL-1 receptor antagonist) improves beta cell survival (Ardestani et al., 2011). IL-1β production in the pancreas is likely mediated by NLRP3 inflammasome-dependent activation of caspase-1 (Youm et al., 2011) as also outlined in Figure 1. In support of this concept, mice that lack NLRP3 inflammasome components (*Nlrp3, Asc*) have increased pancreatic islet size in response to chronic high-fat diet, resulting in increased insulin response to glucose challenge despite improvements in peripheral insulin sensitivity (Youm et al., 2011). Additionally, reduction of Nlrp3 inflammasome activation in chronically obese mice protects the pancreatic beta cells against cell death (Youm et al., 2011). These findings suggest that reduction in Nlrp3 inflamamsome activity may protect the pancreatic islet from caspase-1 mediated inflammatory death. TXNIP may be a crucial mediator connecting beta cell death and inflammasome activation by linking glucotoxicity and ER stress to NLRP3 inflammasome activation (Zhou et al., 2010). Global TXNIP^−/−^ mice recapitulate an insulin sensitive phenotype very similar to that of NLRP3 ablation (Yoshihara et al., 2010). In pancreatic beta cells, TXNIP ablation reduces glucotoxicity, ER Stress and the subsequent inflammatory and apoptotic responses (Zhou et al., 2010; Oslowski et al., 2012). TXNIP serves as a signaling node linking ER stress, IL-1B production and beta cell apoptosis. The consequences of pancreatic beta cell TXNIP and inflammasome activation *in vivo* are unclear because beta cell and myeloid cell specific knockouts have not been used to address this issue. Consistent with the important role of IL-1β in the pancreas, *Il1r1*^−/−^ mice that are deficient in IL-1β signaling exhibit improved insulin-secretion in response to glucose challenge (McGillicuddy et al., 2011). Thus, lowering Nlrp3 inflammasome activation may protect against the transition from insulin-resistance to an overt type 2 diabetic stage by mechanisms that involve protection from loss of insulin-producing beta cells. It is presently unclear whether persistent Nlrp3 inflammasome activation causes the transition from insulin-resistance to islet decompensation and development of overt T2D.

## Therapeutic implications

Initial studies in humans suggest that NLRP3 inflammasome activation in obesity could be important in development and treatment of insulin-resistance and diabetes [Figure 2; (Larsen et al., 2007; Vandanmagsar et al., 2011; Goossens et al., 2012)]. Lee et al. recently published a study on inflammasome activation in blood monocytes isolated from type 2 diabetic, drug naive patients (*n* = 47) and healthy controls (*n* = 57) (Lee et al., 2013). This study determined that both during basal and inflammasome activating conditions (stimulation with free fatty acids, ATP, or urate) blood monocytes from patients with T2D have greater caspase-1 activation and secretion of the caspase-1 activated proteins, IL-1β and IL-18. Inflammasome activation can occur in response to diverse cellular stresses including reactive oxygen species, the unfolded protein response and altered autophagy. In the context of this experiment, hyperglycemia in these T2D patients resulted in elevated ROS production and greater inflammasome activation. Knockdown of ASC or NLRP3 using RNA interference abrogated the response to DAMPs demonstrating specificity to this pathway in T2D patients (Lee et al., 2013). This study provides evidence that the Nlrp3 inflammasome activation in T2D patients contributes toward the chronic pro-inflammatory state.

Gossens et al. designed a study to assess the gene expression of *Nlrp3* and T-cell markers in subcutaneous adipose tissue from lean and obese subjects, and to determine if these genes were associated with glucose homeostasis measured by the hyperinsulinemic-euglycemic clamp (Goossens et al., 2012). Obese subjects had increased body weight, body fat%, adipocyte diameter, fasting glucose, and insulin and glucose infusion rate during the hyperinsulinemic-euglycemic clamp test. The expression of cellular markers of inflammasome activation, i.e., *Nlrp3, caspase-1*, and T cell markers, were positively associated with increased expression of inflammatory genes. Furthermore, *caspase-1* and *Il-18* gene expression, and the ratio of *Tbx21*/*Cd3*ε expression, a marker of pro-inflammatory T-helper 1 cells, were negatively correlated with glucose infusion rate. Consistent with relevance of this pathway in diabetes treatment, *Il-1*β, *Nlrp3*, and *Asc* gene expression is reduced after 1 year of weight loss in obese type 2 diabetic patients, and these gene expression changes were positively correlated with improvements in glycemia (Vandanmagsar et al., 2011). Such associations are further supported by evidence from human adipose tissue explants that high glucose levels induce proinflammatory gene expression (*Il-6, Il-8*, and *Il-1*β), increase intracellular pro-IL1β and secretion of bioactive IL-1β (Koenen et al., 2011). Taken together, these studies indicate that the NLRP3 inflammasome components are expressed in human adipose tissue, are responsive to high glucose concentrations, and associate with markers of glycemia.

Given the key role of the inflammasome in possibly mediating many of the factors associated with progression to T2D, and in control of the condition, it is imperative to test interventions that may favorably interdict on this system. In humans, there has been interest in a specific IL-1 receptor antagonist. Anakinra, IL-1 receptor antagonist, competes with IL-1β for binding to IL1R1 and has received attention as an agent that may have efficacy on glycemic control. Larsen et al. conducted a double-blind, parallel-group trial in which anakinra (*n* = 34) or placebo (*n* = 33) was administered subcutaneously once/day for 13 weeks (Larsen et al., 2007). The study population were patients with T2D, >27 body mass index, glycated hemoglobin >7.5% and had no change in medication type or dose over the course of the study. As expected, subjects in the anakinra treatment group had increased circulating IL-1RA concentration compared to placebo, 1256 ± 958 and 0.6 ± 0.4 μg/L, respectively. Anakinra treatment resulted in significant reduction in glycated hemoglobin % measured by the difference between baseline and 4 or 13 weeks in the treatment versus placebo groups (Larsen et al., 2007). As reported, at 13 weeks, in the anakinra group, the glycated hemoglobin level was 0.46% points lower than in the placebo group (*P* = 0.03).

To explain changes in glycated hemoglobin concentration, beta-cell secretory function and insulin sensitivity were measured at 13 weeks. Anakinra treatment lowered the proinsulin:insulin ratio and increased C-peptide concentration in response to oral or intravenous glucose, all measures were presented as change from baseline. Thus, anakinra treatment increased circulating IL-1RA and the insulin secretory capacity of the pancreas, but had no effect on insulin sensitivity. In agreement with no effect on insulin action, insulin-regulated gene expression in skeletal muscle, serum adipokine levels, and body-mass index were found to be similar in the two study groups. Similar results were achieved from a study on patients with prediabetes that showed enhanced beta-cell function during OGTT in response to anakinra treatment, in the absence of increased insulin sensitivity (van Asseldonk et al., 2011). Blockade of IL-1 with anakinra does improve glycemia and beta-cell secretory function and reduces markers of systemic inflammation.

A follow up study on the diabetics from the Larsen et al. was performed after the initial anakinra treatment was removed. Interestingly, this relatively short term (13 weeks) anakinra treatment had long term effects after the treatment was removed. Thirty-nine weeks after the treatment, the anakinra treated group had an improved blood proinsulin:insulin ratio, decreased C-reactive protein and IL-6 concentrations, but no differences in hemoglobin A1c (Larsen et al., 2009). Within this study there were responders and non-responders. Responders had lower starting serum IL-1RA concentrations and a higher group frequency of a specific gene polymorphism (SNP rs4251961 allele C), which is associated with low circulating IL-1RA (Larsen et al., 2009), indicating that IL-1RA may be beneficial for only specific populations. Reduced IL-6 concentrations may also be responsible for a portion of improvements of pancreatic function with anakinra treatment in that study. A trial utilizing the IL-6 monoclonal antibody Actemra (tocilizumab) during T2D could test this hypothesis.

In summary, the findings thus far indicated that IL-1 blockade with specific agents induces improvement of pancreatic insulin secretory function and reduces markers of systemic inflammation lasting 39 weeks after treatment withdrawal. Given these results, it is of interest to speculate that IL-1β specific monoclonal antibodies will have beneficial effects in T2D. Monoclonal antibodies directed against IL-1β may be favorable compared with anakinra due to the short half-life of anakinra, which requires daily injections, and specificity to IL-1β. While anakinra antagonizes IL-1R1 signaling by both IL-1β and IL-1α, neutralization of IL-1β through monoclonal antibodies may be favorable because IL-1α signaling still remains. Intact, IL-1α signaling may also reduce the efficacy of monoclonal antibodies because inflammasome activators can cause the secretion of IL-1α in addition to IL-1β (Gross et al., 2012). Proof of concept for monoclonal antibodies has been obtained. In a phase 1 study, gevokizumab (XOMA 052), a monoclonal antibody against IL-1β, showed beneficial effects on glycemic control and beta-cell function. Specifically, studies were conducted that evaluated single intravenous infusion or subcutaneous injection of placebo or IL-1β targeted monoclonal antibody at 0.01–3.0 or 0.03–0.3 mg/kg, respectively. One month after antibody administration, there were no significant differences in HbA1c, but at 2 and 3 months a significant reduction was noted in the intermediate doses (0.03–1.0 mg/kg) compared to the combined low dose (0.01 mg/kg) and placebo group (Cavelti-Weder et al., 2012). As expected, intravenous administration of gevokizumab resulted in greater circulating antibody concentrations than subcutaneous administration. Subcutaneous delivery of the 0.03 and 0.3 mg/kg antibody doses at day 0, 14, and 28 did not result in a substantial improvement over a single administration at day 0 alone. It may be that microvascular damage that occurs during T2D is limiting tissue blood perfusion and for this reason the circulating antibody concentrations do not reflect the increased number of doses. A phase 2 trial was done using gevokizumab in 421 patients, but beneficial effects on glycated hemoglobin and glycemia were not observed, while reduction in C-reactive protein was observed (DeGuzman, 2011). The capacity of gevokizumab to improve glucose homeostasis is currently unclear, but like anakinra it may be effective in specific populations. As indicated in the study by Larsen et al., there may be a subset of the population that responds more favorably to IL-1β targeted therapy. The stronger effects of anakinra could be due to inhibition of both IL-1β and IL-1α signaling. It is important to recognize that inflammation is an ongoing process and that IL-1β targeted therapies will reduce inflammation at the time of administration, but prior inflammatory damage incurred during the lifespan may not be readily reversible. Thus, the timing of the intervention may be key to IL-1β targeted therapy and prevention of pancreatic damage may be more useful than treatment of chronic pancreatic inflammation. Importantly, given the NLRP3 inflammasome is an upstream activator of caspase-1 and IL-1, the inhibition of exaggerated NLRP3 inflammasome activation by future drugs may have better therapeutic outcomes in T2D.

Another intriguing question is whether any of the currently available agents we have at our disposal for use in T2D have any effect on the inflammasome. The cellular energy sensor, AMPK, is known to influence caspase-1 activation. As discussed earlier, the NLRP3 inflammasome is activated by fatty acids, notably palmitate, but this effect is blocked by administration of AICAR, an AMPK agonist. Metformin is a known AMPK activator and 2m of metformin treatment reduces NLRP3 inflammasome activation in peripheral blood mononuclear cells from type 2 diabetic patients. In addition to metformin, the sulfonylurea glyburide has been identified as an NLRP3 inflammasome inhibitor, while other sulfonylurea drugs do not inhibit inflammasome activation (Lamkanfi et al., 2009). This is an interesting finding considering the concerns hypoglycemia, weight gain, and secondary failures with use of sulfonylureas. In certain circumstances, particularly sepsis, glyburide can act as an anti-inflammatory and is associated with reduce mortality in lipopolysaccharide challenged rodents, and observationally in type 2 diabetics with melioidosis compared to patients with no diabetes (Lamkanfi et al., 2009; Koh et al., 2011). This effect was limited to glyburide treatment and was not associated with either metformin or insulin treatment. It is presently unclear if the use of glyburide at doses lower than those typically used clinically will have positive effects on beta cell survival and this question may deserve a more thorough investigation. Compared to IL-1 targeted therapies, glyburide may provide the additional benefit of inhibiting the NLRP3 inflammasome and cleavage of many caspase-1 substrates. It would be useful to directly assess the effect of low-dose glyburide on markers of inflammasome activation in diabetics or pre-diabetics. As discussed above, its effects may be more useful for the prevention of pancreatic damage rather than an intervention after damage has already occurred.

Because IL-1 is important for innate immune signaling, blocking its action with IL-1RA could have negative effects on the immune response. In the study by Larsen et al., there were minimal adverse effects of anakinra, with the predominant negative effect being an increase in injection site reactions (Larsen et al., 2007). In a larger study of anakinra treatment for rheumatoid arthritis there was no increase in adverse events besides injection site reactions (Nuki et al., 2002). IL-1 neutralization may provide a path to improving pancreatic function, with minimal adverse effects. However, improvements in drugs targeting this pathway are required because of the modest effects that have been seen thus far.

## Conclusion

IL-1β is an influential immune modulator. Uncontrolled activation of the NLRP3 inflammasome during disease states and increased IL-1β synthesis, secretion, and signaling can lead to inflammatory disease. Initial clinical studies that dampen IL-1β signaling have shown promise in controlling diabetes but detailed evidence to support use of IL-1 neutralizing antibodies in diabetic patients has thus far not yielded successful results. Notably, once cleaved, caspase-1 can regulate the activity of several proteins other than just IL-1β and IL-18 (Agard et al., 2010). New data shows that high-fat diet feeding-induced caspase-1 can deactivate Sirt1 and leads to insulin-resistance (Chalkiadaki and Guarente, 2012). Activation of Sirt1 by resveratrol and other small molecules improves glycemic control (Lagouge et al., 2006; Feige et al., 2008). Thus given inflammasome activation can impair several metabolically relevant signaling proteins such as Sirt1, additional studies are required to test whether specific inflammasome or caspase-1 inhibitors offer better therapeutic alternatives than IL-1β inhibition as treatment for diabetes. Considering that the pancreatic damage that occurs during diabetes is an ongoing inflammatory process that may not be readily reversible, an early preventive strategy targeting NLRP3 inflammasome may prove useful in diabetes and its complications.

### Conflict of interest statement

The authors declare that the research was conducted in the absence of any commercial or financial relationships that could be construed as a potential conflict of interest.
